# Tuberculosis presentation and outcomes in older Hispanic adults from Tamaulipas, Mexico

**DOI:** 10.1097/MD.0000000000035458

**Published:** 2023-10-13

**Authors:** Belinda A. Medrano, Miryoung Lee, Gretchen Gemeinhardt, Javier E. Rodríguez-Herrera, Moncerrato García-Viveros, Blanca I. Restrepo

**Affiliations:** a Department of Epidemiology, Human Genetics and Environmental Sciences, School of Public Health, University of Texas Health Science Center at Houston, Brownsville campus, Brownsville, TX, USA; b Department of Management, Policy and Community Health, School of Public Health, University of Texas Health Science Center at Houston, Houston, TX, USA; c Secretaría de Salud de Tamaulipas, Tamaulipas, México, USA; d Population Health Program, Texas Biomedical Research Institute, San Antonio, TX, USA; e School of Medicine, South Texas Diabetes and Obesity Institute, University of Texas Rio Grande Valley, Edinburg, TX, USA.

**Keywords:** BMI, death, diabetes, gerontology, Hispanics, older adults, older people, social support, TB, treatment outcomes, tuberculosis

## Abstract

Older people are at high risk of developing and dying from pulmonary infections like tuberculosis (TB), but there are few studies among them, particularly in Hispanics. To address these gaps, we sought to identify host factors associated with TB and adverse treatment outcomes in older Hispanics by conducting a cross-sectional study of TB surveillance data from Tamaulipas, Mexico (2006–2013; n = 8381). Multivariable logistic regressions were assessed for older adults (OA ≥65 years) when compared to young (YA, 18–39 years) and middle-aged adults (40–64 years). We found that the OA had features associated with a less complicated TB (e.g., lower prevalence of extra-pulmonary TB and less likely to abandon treatment or have drug resistant TB), and yet, were more likely to die during TB treatment (adj-OR 3.9, 95% 2.5, 5.25). Among the OA, excess alcohol use and low body mass index increased their odds of death during TB treatment, while a higher number of reported contacts (social support) was protective. Diabetes was not associated with adverse outcomes in OA. Although older age is a predictor of death during TB disease, OA are not prioritized by the World Health Organization for latent TB infection screening and treatment during contact investigations. With safer, short-course latent TB infection treatment available, we propose the inclusion of OA as a high-risk group in latent TB management guidelines.

## 1. Introduction

*Mycobacterium tuberculosis* is a bacterial disease that has infected a quarter of the world population with non-symptomatic, latent infection or progressed to active tuberculosis (TB) disease. In 2020, an estimated 9.9 million people developed TB and 1.5 million died.^[1]^ The incidence of TB disease is greatest in ages 25 to 54 years old, worldwide. However, the World Health Organization (WHO) regions of the Eastern Mediterranean, South-East Asia, Western Pacific, and the Americas already have a significant burden of TB cases in older adults (OA).^[2–5]^ For example, Taiwan is anticipating that nearly 80% of their new TB cases will be in OA by 2035.^[6]^ In the US, adults 65 years or older had the highest TB incidence of any age group at 4.0 cases per 100,000 in 2021.^[4]^ This is relevant as the global older adult population grows from 1 out of 11 in 2019 to 1 in 6 by 2050.^[7]^ Older adults face additional risks of TB reactivation and infection compared to younger adults, such as compromised immunity, multiple chronic comorbid conditions, and increased TB exposures throughout their lifetime or due to clustering in nursing homes.^[8–10]^ TB and general mortality is highest in OA.^[2,11]^ An aging population, longer life expectancy, and reactivation of TB disease in OA pose challenges to meeting the WHO “End TB Strategy” goals of a 90% and 95% reduction in TB incidence and deaths, respectively, by 2035.^[1,9,11–13]^

Despite their high vulnerability, TB risks in OA are understudied, and the available literature is mostly from Asian populations.^[3,14]^ This contrasts with fewer studies in Hispanics.^[15–17]^ We have begun addressing this gap among Hispanics in the Texas-Mexico border. Secondary analysis of TB surveillance data from Mexico suggests that OA comprise about 10% of all adult TB patients,^[18]^ and our prospective studies are revealing unique features of TB pathogenesis in OA (e.g., diabetes, Bacille Calmette-Guiren [BCG] vaccination).^[16,17,19]^ Thus, further studies in older Latin American Hispanics are warranted given that risk factors for TB such as poverty, migration, homelessness, incarceration, and diabetes have increased in this region in the last decade.^[20,21]^

Given the impact of older age on TB elimination efforts, it is important to study the older adult TB group with the goals of improving patient outcomes and preventing additional TB cases. To address this knowledge gap, we aimed to identify sociodemographic and medical risk factors and secular trends for TB in older Hispanic adults from the state of Tamaulipas in northeastern Mexico. We also sought to identify the risk factors associated with adverse TB outcomes in older age.

## 2. Materials and methods

### 2.1. Study population

We conducted a secondary analysis of de-identified TB surveillance data on adults ages 18 to 99 years with a new TB diagnosis and who were reported to the state of Tamaulipas, Mexico between 2006 and 2013. The state TB program surveillance dataset was described previously.^[18]^ Briefly, the Tamaulipas TB surveillance dataset was comprised of 12,748 TB patients enrolled between 2005 and 2013, and we excluded repeat episodes from the same subject (n = 1827), individuals <18 years old (n = 624), recurring TB cases (n = 777), and individuals enrolled in 2005 who had poor data quality (n = 1089). This left 8431 new adult TB patients.^[18]^ For this study, we further excluded 43 individuals identified as having a previous TB episode and 7 with missing data on TB treatment outcomes, for a final sample size of 8381 TB patients.

### 2.2. Participant characteristics

We compared OA (65 years. or older) to young adults (18–39 years, YA) or middle-aged adults (40–64 years, MAA). Sociodemographic variables included age, sex, education, BCG vaccination at birth, based on the presence of a BCG scar, self-reported excess alcohol use, intravenous (IV) drug use, and low body mass index (BMI < 18.5 kg/m^2^ at the time of TB diagnosis). Comorbidities included chronic obstructive pulmonary disease (COPD), self-reported diabetes, and self-reported or laboratory-confirmed human immunodeficiency virus (HIV). Variables related to TB at the time of diagnosis included disease location (extrapulmonary or pulmonary), positive acid-fast bacilli (AFB) on sputum smears, TB diagnosis method [culture confirmation by isolation of *M tuberculosis*, positive AFB smears only, or in their absence, a combination of clinical, epidemiological, radiological and/or histopathological findings], and the number of close contacts. In Mexico, drug susceptibility testing is limited to patients with high suspicion of drug-resistant TB with results available for isoniazid (INH), rifampin, pyrazinamide, streptomycin, and ethambutol. *M tuberculosis* was classified as drug resistant tuberculosis (DR-TB) if resistant to any of the 5 TB drugs and multi-drug resistant when at least INH and rifampin were resistant. The adverse outcomes of interest included TB treatment failure (positive AFB smears after 5 months of TB treatment), abandoned treatment (lost during follow-up for more than 3 months), and death, due to any cause, during TB treatment.

### 2.3. Statistical analyses

Complete-case analysis was utilized in this study. Two-group comparisons of binary variables were analyzed by Pearson chi-square or Fisher exact test and the Student *t* test was used for mean comparisons between 2 groups. Increasing or decreasing trends across age groups or across time were established by the score test for the trend of odds for binary variables. To conduct comparisons between-group, trends across age groups, and trends across time for polytomous and numerical variables, we used the nptrend command in STATA, which is a nonparametric test for trends across ordered groups (an extension of the Wilcoxon rank-sum test). For multiple logistic regression models, we used backward selection with consideration of variables with *P* values < .20 in bivariable analysis, and keeping age and sex as key sociodemographics in final models. Effect modification variables for age and each predictor variable were included in simple logistic regression models for adverse outcomes, to determine if age modifies the observed effect of each predictor variable. Statistical significance was set at Type I error (alpha) level < .05. Data analysis was performed using STATA IC (Stata Corp LLC, College Station, TX) 14.

### 2.4. Ethical compliance

Patient data was deidentified and the protocol was approved from the Internal Review Board from UTHealth Houston (HSC-SPH-15-0489) and the Secretaría de Salud de Tamaulipas (076/2015/CEI).

## 3. Results

### 3.1. Sociodemographics and comorbidities in the older adults

The characteristics of the 8381 TB patients for analysis are described in Table 1. Their mean age was 43 years (standard deviation [SD] 16.6; range 18–97). Males comprised 65.5% of all TB patients. Less than half (42.2%) had a high school degree or higher education, 3-fourths (76.2%) had a history of BCG vaccination, 5.6% reported excess alcohol use, 0.5% were IV drug users, and 8.3% had low BMI. The most prevalent comorbidity was diabetes (25.2%), followed by HIV (5.3%), and COPD (0.9%).

**Table 1:** Descriptive characteristics of TB patients by age group, Tamaulipas 2006–2013.

	All adults	YA	MAA	OA	OA vs	OA vs	Trend across
	(≥ 18 yr)	(18–39 yr)	(40–64 yr)	(≥65 yr)	YA	MAA	Age groups
Total n (row %)	8381 (100)	3854 (46.0)	3492 (41.7)	1035 (12.3)	*P* value	*P* value	*P* value†
Sociodemographic characteristics							
Age (mean, SD)	43 (16.6)	28 (6.2)	50 (6.9)	73 (6.5)			
Male sex	5493 (65.5)	2554 (66.3)	2287 (65.5)	652 (63.0)	.049	.139	.068
High school or higher (n = 8040)	3395 (42.2)	2093 (56.9)	1194 (35.5)	108 (10.9)	<.001	<.001	↓ <.001
BCG vaccination (n = 7814)	5955 (76.2)	3052 (83.6)	2453 (75.7)	450 (48.8)	<.001	<.001	↓ <.001
Excess alcohol	473 (5.6)	198 (5.1)	236 (6.8)	39 (3.8)	.069	<.001	.995
IV drug use	39 (0.5)	30 (0.8)	8 (0.2)	1 (0.1)	.013	.694	↓ <.001
Low BMI	696 (8.3)	360 (9.3)	229 (6.6)	107 (10.3)	.333	<.001	.275
Comorbidities							
COPD	72 (0.9)	5 (0.1)	25 (0.7)	42 (4.1)	<.001	<.001	↑ <.001
Diabetes	2111 (25.2)	386 (10.0)	1395 (40.0)	330 (31.9)	<.001	<.001	↑ <.001
HIV	442 (5.3)	297 (7.7)	140 (4.0)	5 (0.5)	<.001	<.001	↓ <.001
TB-related characteristics							
TB location (n = 8380)							
Extrapulmonary TB	670 (8.0)	362 (9.4)	246 (7.0)	62 (6.0)	.001	.237	↓ <.001
Pulmonary TB	7710 (92.0)	3491 (90.6)	3246 (93.0)	973 (94.0)			
Positive AFB Smear (n = 7683)	6537 (85.1)	2934 (83.7)	2781 (86.1)	822 (86.9)	.016	.520	↑.002
TB diagnosis type							
Culture confirmation	571 (6.8)	308 (8.0)	206 (5.9)	57 (5.5)	.017	.195	.794
AFB Smear only	6631 (79.1)	2957 (76.7)	2841 (81.4)	833 (80.5)			
Clinical-epidemiological	1145 (13.7)	571 (14.8)	436 (12.5)	138 (13.3)			
Missing	34 (0.4)	18 (0.5)	9 (0.3)	7 (0.7)			
No. of contacts (mean, SD) (n = 8103)	3.7 (2.6)	3.7 (2.4)	3.6 (2.5)	4.1 (3.1)	<.001	<.001	.220
Adverse treatment outcomes	1252 (14.9)	592 (15.4)	452 (12.9)	208 (20.1)	<.001	<.001	.097
Treatment failure	156 (1.9)	64 (1.7)	69 (2.0)	23 (2.2)	.225	.622	.176
Abandoned treatment	579 (6.9)	356 (9.2)	179 (5.1)	44 (4.3)	<.001	.253	↓ <.001
Died	517 (6.2)	172 (4.5)	204 (5.8)	141 (13.6)	<.001	<.001	↑ <.001

Data expressed as number (column %) for categorical variables unless specified, and mean (standard deviation) for age and number of contacts. Analysis of each variable includes all 8381 TB patients except when indicated in parenthesis.

Clinical-epidemiological = diagnosis based on signs and symptoms, epidemiology, radiological and/or histological findings.

AFB = acid-fast bacilli, BCG = Bacille Calmette-Guiren, BMI = body mass index, COPD = chronic obstructive pulmonary disease, HIV = human immunodeficiency virus, IV = intravenous, MAA = middle-aged adults, OA = older adults SD = standard deviation, TB = tuberculosis, YA = young adults.

† Trend direction with respect to older age is indicated by arrows preceding significant trend *P* values.

The distribution of TB patients by age group was: 46% in YA, 42% in MAA, and 12% in OA. Mean ages for YA, MAA, and OA TB patient groups were 28 (SD 6.2), 50 (SD 6.9), and 73 (SD 6.5) years, respectively. The OA differed from the 2 younger age groups in their lower level of education (high school degree or higher in 10.9% in OA vs 56.9% in YA or 35.5% in MAA), BCG vaccination (48.8% in OA vs 83.6% in YA and 75.7% in MAA), and IV drug use (0.1% in OA vs 0.8% in YA). Accordingly, significant trends with older age (from YA to MAA to OA) included a decrease in education level, BCG vaccination rates, and IV drug use, trend *P* < .001 for all. Low BMI was highest in the OA (10.3%) followed by YA (9.3%) and MAA (6.6%).

For comorbidities, COPD was more common among the OA (4.1% in OA vs 0.1% in YA and 0.7% in MAA; *P* < .001 for increasing trend with older age). Diabetes increased with older age (trend *P* < .001), although MAA had the highest prevalence (40%), with the OA having 31.9% and YA 10% (*P* < .001). HIV prevalence was lowest in the OA (0.5% in OA vs 7.7% YA and 4.0% in MAA; *P* < .001 for decreasing trend with older age).

### 3.2. TB-related characteristics

Most patients had pulmonary TB (92%) and a positive AFB smear at the time of diagnosis (85.1%; Table 1). Cultures are not routinely performed in Tamaulipas, and hence, the diagnosis was mostly based on smear results (79.1%), with only 6.8% being culture-confirmed. Each TB patient had an average of 3.7 close contacts. When compared to YA, the OA had less extra-pulmonary TB (OA 6.0% vs YA 9.4%; decreasing trend *P* < .001) or culture-confirmed TB (OA 5.5% vs YA 8.0%). The OA had the highest mean number of close contacts (OA 4.1 vs 3.7 in YA or 3.6 in MAA).

### 3.3. TB drug resistance

Drug susceptibility testing was only available for 1165 (13.9%) TB patients, with more testing in YA (15.4%) and MAA (14.2%) vs. the OA (7.3%; trend *P* < .001; Table 2). For primary analysis, we assumed that the absence of susceptibility results indicated drug-susceptible TB since testing is prompted when DR-TB is suspected (Table 2). We found that DR-TB decreased with age (trend *P* = .030). Among all age groups, the highest monoresistance was for INH (1.2%), and across age groups, only INH monoresistance showed a decreasing trend with age (*P* = .002). Multi-DR-TB occurred in 0.6% of all patients and did not differ across age groups. Similar analysis but limited to isolates tested for drug resistance provided similar findings (Table S1, http://links.lww.com/MD/K232).

**Table 2:** Tuberculosis (TB) drug resistance prevalence by age group.

	All adults	YA	MAA	OA	Trend across age groups
	(≥18 yr)	(18–39 yr)	(40–64 yr)	(≥65 yr)	*P* value*^,^†
Total n	8381	3854	3492	1035	
Tested	1165 (13.9)	593 (15.4)	496 (14.2)	76 (7.3)	↓ <.001
DR-TB	257 (3.1)	134 (3.5)	99 (2.8)	24 (2.3)	↓.030
INH	99 (1.2)	57 (1.5)	39 (1.1)	3 (0.3)	↓.002
RIF	6 (0.1)	4 (0.1)	2 (0.1)	0 (0)	.238
PZA	6 (0.1)	2 (0.1)	3 (0.1)	1 (0.1)	.544
STR	36 (0.4)	12 (0.3)	17 (0.5)	7 (0.7)	.083
EMB	4 (0.1)	3 (0.1)	0 (0)	1 (0.1)	.633
MDR-TB	54 (0.6)	26 (0.7)	22 (0.6)	6 (0.6)	.715

Data expressed as n (column %).

Mono-resistance is listed for individual antibiotics: INH = isoniazid, RIF = rifampin, PZA = pyrazinamide, STR = streptomycin, EMB = ethambutol. MDR-TB = multi-drug resistant TB defined as resistant to at least INH and RIF.

DR-TB = drug resistant tuberculosis, MAA = middle-aged adults, OA = older adults, Tested = susceptibility testing documented, YA = young adults.

* Score test for trend of odds was calculated across the 3 age groups.

† Trend direction with respect to older age is indicated by arrows preceding significant trend *P* values.

### 3.4. Adverse TB treatment outcomes

Among all patients, 14.9% had one of the following adverse outcomes: treatment failure (1.9%), abandoned treatment (6.9%), or death during TB treatment (6.2%), Table 1. Adverse TB treatment outcomes were highest among the OA (20.1%) followed by YA (15.4%) and MAA (12.9%) groups. Treatment failure did not differ between age groups, but the OA group was less likely to abandon treatment (4.3%) when compared to YA (9.2%; *P* < .001) with a decreasing trend with older age (trend *P* < .001). In contrast, the OA were more likely to die during TB treatment (13.6%) when compared to YA (4.5%) or MAA (5.8%), with an increasing trend with older age (trend *P* < .001).

For multivariable analysis, we examined whether old age was independently associated with adverse TB treatment outcomes with YA as the reference group. IV drug use and HIV were excluded as predictor variables due to their low prevalence in the OA group (Table 3). Age groups were not associated with treatment failure. Instead, among all age groups, adjusted models showed that treatment failure was higher for males, although statistical significance was not reached (adjusted odds ratio [adj. OR] 1.45, 95% CI 0.99, 2.10), those with positive AFB smears (adj. OR 1.97, 95% CI 1.03, 3.79) or DR-TB, (adj. OR 13.85, 95% CI 9.42, 20.37). Higher education was protective against treatment failure (adj. OR 0.69, 95% CI 0.48, 0.99).

**Table 3:** Multivariable analyses for predicting treatment failure, abandoned treatment, and died during TB treatment.

Predictor variables	Treatment failure		Abandoned treatment		Died during treatment	
	adj. OR	95% CI	adj. OR	95% CI	adj. OR	95% CI
Age group						
YA (18–39 yr)	1.00		1.00		1.00	
MAA (40–64 yr)	1.23	0.85, 1.77	0.49	0.40, 0.61	1.62	1.25, 2.09
OA (≥65 yr)	1.39	0.83, 2.33	0.41	0.28, 0.59	3.90	2.90, 5.25
Male sex	1.45	0.99, 2.10	2.09	1.65, 2.66	1.59	1.24, 2.04
High school or higher	0.69	0.48, 0.99	0.74	0.60, 0.90		
BCG vaccination					0.67	0.53, 0.85
Excess alcohol			1.44	1.03, 2.01	1.66	1.15, 2.39
Low BMI			1.59	1.18, 2.13	2.14	1.60, 2.87
Diabetes					0.59	0.44, 0.78
Extrapulmonary TB			0.51	0.30, 0.87	1.94	1.29, 2.91
Positive AFB Smear	1.97	1.03, 3.79	0.63	0.49, 0.82	0.63	0.47, 0.84
No. of contacts			0.89	0.85, 0.93		
DR-TB	13.85	9.42, 20.37				

Full regression models include age group, sex, education, BCG vaccination, excess alcohol use, low BMI, COPD, diabetes, TB disease location, positive AFB smear, number of contacts, and DR-TB. All reduced models (shown) include age and sex plus predictor variables with a significance level < 0.05.

adj. OR = adjusted odds ratio, AFB = acid-fast bacilli, BCG = Bacille Calmette-Guiren, BMI = body mass index, CI = confidence intervals, DR-TB = drug resistant tuberculosis, MAA = middle-aged adults, OA = older adults, TB = tuberculosis, YA = young adults.

Abandoning treatment was 59% lower in the OA (adj. OR 0.41, 95% CI 0.28, 0.59) when compared to YA (Table 3). Among all TB patients, the factors associated with higher odds of abandoning treatment were male sex (adj. OR 2.09, 95% CI 1.65, 2.66), excess alcohol use (adj. OR 1.44, 95% CI 1.03, 2.01), and low BMI (adj. OR 1.59, 95% CI 1.18, 2.13). Protective factors against abandoning treatment included high school or higher education (adj. OR 0.74, 95% CI 0.60, 0.90), having extrapulmonary disease (adj. OR 0.51, 95% CI 0.30, 0.87), and positive AFB smear at diagnosis (adj. OR 0.63, 95% CI 0.49, 0.82). The odds of abandoning treatment decreased by 11% (adj. OR 0.89, 95% CI 0.85, 0.93) for each additional close contact reported by a TB patient.

Death during TB treatment was 4-fold higher with old age (OA vs YA: adj. OR 3.90, 95% CI 2.90, 5.25; Table 3). Among all TB patients, the odds of death during TB treatment were also higher for MAA (adj. OR 1.62, 95% CI 1.25, 2.09), males (adj. OR 1.59, 95% CI 1.24, 2.04), excess alcohol users (adj. OR 1.66, 95% CI 1.15, 2.39), those with low BMI (adj. OR 2.14, 95% CI 1.60, 2.87), and extrapulmonary disease (adj. OR 1.94, 95% CI 1.29, 2.91). Death during TB treatment were lower for patients with BCG vaccination (adj. OR 0.67, 95% CI 0.53, 0.85), diabetes (adj. OR 0.59, 95% CI 0.44, 0.78), and a positive AFB smear (adj. OR 0.63, 95% CI 0.47, 0.84), respectively.

**Figure 1.**
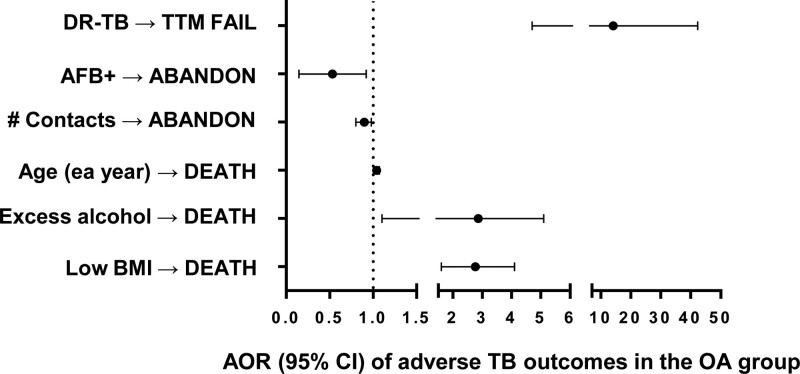
Adjusted odds ratio of adverse tuberculosis (TB) outcomes in older adults (OA) group. Multivariable analysis was conducted among OA TB patients. The initial model considered all variables with *P* ≤ .20 in bivariable analysis, followed by backwards selection leaving only age, sex and any variable with *P* ≤ .05 in the final model. The exposure variables with a significant association are shown. Adverse outcomes listed in capital letters. TTM FAIL = treatment failure, ABANDON = abandon treatment, DEATH = death from any cause during TB episode, AOR = adjusted odds ratio, OA = individuals 65 yr or older.

Among the OA age group alone, we identified independent predictors of adverse outcomes (Fig. 1; Table S2, http://links.lww.com/MD/K233). Treatment failure was 14 times higher in patients with DR-TB (adj. OR 14.10, 95% CI 4.69, 42.32). Abandoning treatment decreased by 56% for those with a positive AFB smear and by 15% for each close contact. The odds of death during TB treatment increased by more than 2-fold in those with excess alcohol use or low BMI. Male sex was not associated with any adverse outcome in the OA (Table S2, http://links.lww.com/MD/K233), despite its relationship with treatment failure, abandoning treatment, and death during TB treatment in all age groups (Table 3).

Finally, we evaluated if among all TB patients, age would modify the associations between host factors and adverse TB treatment outcomes. However, multivariable models with additional interaction terms for age did not show significant associations (Table S3, http://links.lww.com/MD/K234).

### 3.5. Trends over the eight-year period in the OA

We evaluated if there were trends over time (2006–2013) in the prevalence or characteristics of the OA group. Among all TB patients, the proportion of OA remained similar across the years (range 11.2–13.8%; *P* = .53; Table S4, http://links.lww.com/MD/K235). Among the OA group only, there was an increasing trend in education level (*P* < .001), BCG vaccination (P.001), diabetes (*P* = .01), extrapulmonary TB (*P* = .005) and DR-TB (*P* = .002; Tables 4 and S4, http://links.lww.com/MD/K235). We also evaluated trends over time for younger adults (Table 4) and found that unlike the OA, the YA and MAA had a reduction in excess alcohol use, low BMI, and abandoning of TB treatment, and that the YA had a decreasing trend in death during TB treatment.

**Table 4:** Trends across time (2006–2013) in sociodemographic and clinical characteristics of TB patients by age groups.*^,^†

	All adults	YAA	MAA	OA
	(≥18 yr)	(18–39 yr)	(40–64 yr)	(≥65 yr)
	8381 (100%)	3854 (46.0%)	3492 (41.7%)	1035 (12.3%)
Sociodemographic characteristics				
Male sex	↓.043	.070	.218	.982
High school or higher education (n = 8040)	↑<.001	↑<.001	↑<.001	↑<.001
BCG vaccination (n = 7814)	↑<.001	↑<.001	↑<.001	↑.001
Excess alcohol	↓<.001	↓<.001	↓<.001	.168
Low BMI	↓<.001	↓.004	↓<.001	.158
Comorbidities				
COPD	.663	.065	.844	.791
Diabetes	↑<.001	↑.028	↑.009	↑.010
TB-related characteristics				
Extrapulmonary TB (n = 8380)	↑<.001	.060	↑.022	↑.005
Positive AFB Smear (n = 7683)	↑<.001	↑<.001	↑<.001	.179
DR-TB	↑<.001	↑<.001	↑<.001	↑.002
No. of contacts (n = 8103)	↑.017	↑.007	.910	.090
All adverse treatment outcomes	↓.001	↓<.001	.256	.308
Treatment failure	.976	.318	.539	.525
Abandoned treatment	↓<.001	↓.003	↓.028	.789
Died	.278	↓.004	.934	.283

N = 8381 for each variable totals unless indicated in parenthesis. DR-TB = resistant to any of the TB drugs tested (i.e., isoniazid, rifampin, pyrazinamide, streptomycin, ethambutol).

AFB = acid-fast bacilli, BCG = Bacille Calmette-Guiren, BMI = body mass index, COPD = chronic obstructive pulmonary disease, DR-TB = drug resistant tuberculosis, MAA = middle-aged adults, OA = older adults, TB = tuberculosis.

* *P* values shown for the score test for trend of odds for categorical variables and the nonparametric test for trend across ordered groups, for number of contacts.

† Trend direction with respect to older age is indicated by arrows preceding significant trend *P* values.

## 4. Discussion

Despite projections showing a worldwide increase in the proportion of older individuals, studies in the OA are underrepresented when compared to those in younger adults.^[22–24]^ This knowledge gap extends to the OA with TB, especially in the Americas. To address this disparity, we sought to obtain a better understanding of older Hispanic adults with TB using TB surveillance data from the Mexican state of Tamaulipas. The OA comprised 12.3% of all adult TB patients, and over the 8-year study period we observed: an improvement in factors that could be protective for TB (e.g., higher education and BCG vaccination coverage), an increase in factors that favor adverse TB outcomes (e.g., extrapulmonary TB and DR-TB),^[25–29]^ and an increase in diabetes, which had a protective effect on TB outcomes in OA in the current study, as discussed below. The OA differed from younger adults in their higher prevalence of COPD, which could increase their odds of a more severe TB disease, and in having less BCG vaccination and lower education level, which have been associated with higher odds of TB.^[17,30,31]^ However, older age was also associated with factors known to improve their TB prognoses such as less IV drug use, HIV, and extra-pulmonary TB, and abandonment of treatment.^[32–34]^ The distribution of risk factors for TB, therefore, can have variations across all adult age groups and identifying the high-risk profiles in the OA is important for prompt TB suspicion, diagnosis, and treatment initiation, in order to improve their prognosis.

Regardless of a combination of risk and protective factors for TB in the OA in multivariable models, the highest threat to this age group was their higher odds of death of any cause during TB treatment. This finding is consistent with previous studies in OA, and we now report it in our Hispanic TB patients.^[10,35]^ And among the OA group, death during TB treatment was associated with excess alcohol use and low BMI. These factors also contribute to the higher odds of death during TB treatment in all adult age groups.^[1,2,28,36,37]^ In contrast, male sex was a risk factor for death during treatment in YA and MAA TB patients, but not in the OA in our study.

The prevalence of type 2 diabetes increases with age, as observed in this study.^[38,39]^ Diabetes is a risk for TB in adults, including our findings in Mexican Hispanics,^[18,40,41]^ but not in older people.^[16,17]^ Here we evaluated if diabetes would be associated with higher odds of death, but we found the opposite: Diabetes was protective in any age group, and borderline significant among OA (not shown), as reported in Brazil among adults older than 60 years of age.^[42]^ A higher risk of adverse TB treatment outcomes in the presence of diabetes has been reported in previous studies, although not specifically evaluated in older patients.^[10,28,43]^ A possible explanation for our discrepant findings may be the higher prevalence of obesity in diabetes patients. While diabetes is a risk factor for TB, obesity is protective against TB.^[44,45]^ Consistent with this finding, a study in adults from India reported that diabetes offsets the risk of adverse outcomes conferred by low BMI.^[46]^ Further studies are needed to understand the relationship between diabetes, BMI, death in TB patients, and old age. Finally, metformin use as hypoglycemic agent may be protective for TB in diabetes patients,^[47]^ but the use of medications to manage diabetes was not available in our retrospective dataset.

We found that BCG vaccination at birth decreased with older age. This is consistent with the lower prevalence of BCG vaccination in the OA versus younger adults with TB in a prospective research cohort.^[17]^ In that study, BCG vaccination at birth was associated with lower odds of TB, but only in the OA. This finding was intriguing given that BCG is known to protect infants and young children from TB, but not adults.^[1]^ Here we find that BCG vaccination at birth is associated with lower odds of death during TB treatment in all age groups. Our findings are consistent with the association between unfavorable TB treatment outcomes and absence of a BCG scar in a Malaysian population.^[28]^ Together, these findings suggest a possible protective role of BCG with respect to death during TB treatment, although further studies are needed to rule out confounding factors that were not available in the surveillance dataset, such as lower access to healthcare in non-BCG-vaccinated individuals. At the cellular level, BCG vaccination induces epigenetic changes in innate immune cells (e.g., trained immunity) that associate with protection against TB and other microbial infections.^[48]^ Thus, basic science studies are also warranted to help elucidate how BCG vaccination at birth may contribute to TB protection in OA.

We found a decreasing prevalence of extrapulmonary TB with older age, which is consistent with previous studies.^[34,49]^ These age-related differences may be due to a lower prevalence of comorbidities like HIV, when compared to younger TB patients, that favor extrapulmonary disease.^[34,49,50]^

Abandonment of treatment contributes to adverse outcomes but we found that the OA are less likely to abandon TB treatment.^[33,51,52]^ Interestingly, we found an inverse association between the number of TB contacts and odds of abandoning treatment in all age groups, or among OA patients only. Further studies are needed to determine if this finding is consistent with reports indicating that the level and quality of social support received by patients affects their health outcomes and adherence to treatments.^[53–56]^

Patients with drug-resistant TB are more likely to have adverse treatment outcomes.^[1,52,57,58]^ While we found a lower prevalence of DR-TB in the OA, our findings point to the importance of identifying these patients given their higher odds of treatment failure.

Our large sample size, coverage over an 8-year period, and breakdown of age into 3 groups, allowed for an in-depth cross-sectional assessment of unique features in OA TB patients with respect to their sociodemographics, comorbidities, TB characteristics, and treatment outcomes. Limitations included the secondary nature of the data analyses. Diabetes diagnosis and duration of disease, excess alcohol use, and, to some extent, HIV status were self-reported and may be underestimated, although we do not anticipate that there would be a differential underreporting of these host characteristics in the OA when compared to other age groups. Low BMI was reported as a categorical variable with no further granularity to assess the contribution of different levels of body weight or obesity to TB risk or protection in the OA. Smoking is a risk factor for TB and known to be more prevalent in older cohorts, but not reported in this surveillance dataset.^[17]^ DR-TB testing was only evaluated upon suspicion, as in most TB-endemic countries worldwide, and hence, pan-susceptibility was assumed when data was not available. However, analysis limited to patients in whom drug susceptibility was tested provided similar susceptibility trends (Table S1, http://links.lww.com/MD/K232). Abandoning treatment, defined as lost during follow-up for more than 3 months, was presumed to be due to discontinuation of TB treatment, although unreported death cannot be completely ruled out as an unidentified outcome. Finally, TB-specific deaths were not captured in this study, therefore death during TB treatment as an outcome should be interpreted with caution.

## 5. Conclusion

We found that OA do not abandon treatment at higher proportions than younger adult TB patients, which suggests their ability to also comply with latent TB infection (LTBI) treatment. We also identified modifiable sociodemographic factors associated with higher odds of death during TB treatment in the OA, such as excess alcohol use or low BMI. Older adults with new TB or LTBI should be offered education on the risks of excess alcohol use or nutritional support. We also found that abandoning treatment was more likely, regardless of age group, when patients have fewer contacts. Hence, protocols at TB treatment facilities that emphasize the importance of social support, can have a positive impact on treatment success.

Per WHO LTBI management guidelines, TB contact investigations focus on the identification of high-risk groups for TB development. However, these are limited to children below 5 years of age or people living with HIV, and of other adult and child household contacts without mention of the growing older adult population.^[59]^ Our findings call for the need to consider the OA in a similar high-risk category. Progression of LTBI to TB disease can be prevented in OA with safe, short-course LTBI treatments.^[60]^ By studying the older adult TB group separately from the other distinct adult age groups, we will have a better understanding of how to approach the challenges of TB eradication in this understudied and vulnerable population.

## Acknowledgments

We thank the personnel from all the sanitary jurisdictions of the Secretaría de Salud de Tamaulipas who contributed to the collection and recording of data from the TB patients, and Dr Santa Elizabeth Ceballos Liceaga for providing the surveillance datasets for our analysis as coordinator of the Sistema de Vigilancia Epidemiológica de Tuberculosis y Lepra, Dirección General de Epidemiología, Secretaría de Salud de Mexico. We also thank Dr Bassent Abdelbary for generation of the basic dataset used for analysis and guidance for data analysis.

## Author contributions

**Conceptualization:** Belinda A. Medrano, Blanca I. Restrepo.

**Data curation:** Javier E. Rodríguez-Herrera, Moncerrato García-Viveros.

**Formal analysis:** Belinda A. Medrano.

**Funding acquisition:** Blanca I. Restrepo.

**Investigation:** Javier E. Rodríguez-Herrera, Moncerrato García-Viveros, Blanca I. Restrepo.

**Methodology:** Belinda A. Medrano, Miryoung Lee, Gretchen Gemeinhardt, Moncerrato García-Viveros, Blanca I. Restrepo.

**Project administration:** Javier E. Rodríguez-Herrera, Moncerrato García-Viveros, Blanca I. Restrepo.

**Resources:** Blanca I. Restrepo.

**Supervision:** Miryoung Lee, Gretchen Gemeinhardt, Blanca I. Restrepo.

**Validation:** Miryoung Lee, Javier E. Rodríguez-Herrera.

**Writing – original draft:** Belinda A. Medrano.

**Writing – review & editing:** Belinda A. Medrano, Miryoung Lee, Gretchen Gemeinhardt, Javier E. Rodríguez-Herrera, Moncerrato García-Viveros, Blanca I. Restrepo.

## Supplementary Material
