# Supplementary material for: Tuberculosis presentation and outcomes in older Hispanic adults from Tamaulipas, Mexico
Source: Medicine (Baltimore). 2023 Oct 13;102(41):e35458. doi: 10.1097/MD.0000000000035458 (PMC10578661; doi:10.1097/MD.0000000000035458)
Supplement: Supplementary file 1 [file medi-102-e35458-s001.docx]

| **Table S1. TB drugs susceptibility sub-analysis that excludes not tested and unknown from drug resistance calculations** | | | | | |
| --- | --- | --- | --- | --- | --- |
|  | **All Adults** | **YA** | **MAA** | **OA** | Trend |
|  | (≥ 18 y) | (18 - 39 y) | (40 - 64 y) | (≥65 y) | p-value^a,ⴕ^ |
| **INH, n tested** | 1050 | 528 | 458 | 64 |  |
| Resistant | 99 (9.4) | 57 (10.8) | 39 (8.5) | 3 (4.7) |  |
| Susceptible | 951 (90.6) | 471 (89.2) | 419 (91.5) | 61 (95.3) | 0.075 |
| **RIF, n tested** | 1084 | 551 | 465 | 68 |  |
| Resistant | 6 (0.6) | 4 (0.7) | 2 (0.4) | 0 |  |
| Susceptible | 1078 (99.5) | 547 (99.3) | 463 (99.6) | 68 | 0.374 |
| **PZA, n tested** | 680 | 347 | 295 | 38 |  |
| Resistant | 6 (0.9) | 2 (0.6) | 3 (1.0) | 1 (2.6) |  |
| Susceptible | 674 (99.1) | 345 (99.4) | 292 (99.0) | 37 (97.4) | 0.238 |
| **STR, n tested** | 964 | 502 | 405 | 57 |  |
| Resistant | 36 (3.7) | 12 (2.4) | 17 (4.2) | 7 (12.3) |  |
| Susceptible | 928 (96.3) | 490 (97.6) | 388 (95.8) | 50 (87.7) | ↑0.001 |
| **EMB, n tested** | 1094 | 554 | 471 | 69 |  |
| Resistant | 4 (0.4) | 3 (0.5) | 0 | 1 (1.5) |  |
| Susceptible | 1090 (99.6) | 551 (99.5) | 471 | 68 (98.6) | 0.853 |
| **MDR-TB, n tested** | 990 | 488 | 435 | 67 |  |
| Yes | 54 (5.5) | 26 (5.3) | 22 (5.1) | 6 (9.0) |  |
| No | 936 (94.6) | 462 (94.7) | 413 (94.9) | 61 (91.0) | 0.501 |

Note: Data expressed as n (column %). YA=young adults, MAA=middle-aged adults, OA=older adults. Mono-resistance is listed for: INH= isoniazid, RIF= rifampin, PZA= pyrazinamide, STR= streptomycin, EMB= ethambutol. MDR-TB=multi-drug resistant TB defined as resistant to at least INH and RIF.

^a^ Score test for trend of odds was calculated across the three age groups

^ⴕ^ Significant trend p value is preceded by arrow indicating trend direction with respect to older age
