# Supplementary material for: Tuberculosis presentation and outcomes in older Hispanic adults from Tamaulipas, Mexico
Source: Medicine (Baltimore). 2023 Oct 13;102(41):e35458. doi: 10.1097/MD.0000000000035458 (PMC10578661; doi:10.1097/MD.0000000000035458)
Supplement: Supplementary file 2 [file medi-102-e35458-s002.docx]

| **Table S2. Multivariable analyses for predicting treatment failure, abandoned treatment, and died during TB treatment in the OA group** | | | | | | | | | |
| --- | --- | --- | --- | --- | --- | --- | --- | --- | --- |
| **Predictor Variables** | **Treatment Failure** | |  | **Abandoned Treatment** | |  | **Died During Treatment** | |  |
|  | adj. OR | 95% CI |  | adj. OR | 95% CI |  | adj. OR | 95% CI |  |
| Age (y) | 0.98 | 0.91, 1.05 |  | 1.00 | 0.95, 1.06 |  | 1.04 | 1.01, 1.07 |  |
| Male Sex | 1.01 | 0.41, 2.44 |  | 1.40 | 0.68, 2.89 |  | 1.26 | 0.85, 1.86 |  |
| Excess Alcohol |  |  |  |  |  |  | 2.37 | 1.11, 5.06 |  |
| Low BMI |  |  |  |  |  |  | 2.55 | 1.58, 4.11 |  |
| Positive AFB Smear |  |  |  | 0.44 | 0.20, 0.96 |  |  |  |  |
| No. of Contacts |  |  |  | 0.85 | 0.75, 0.98 |  |  |  |  |
| DR-TB | 14.10 | 4.69, 42.32 |  |  |  |  |  |  |  |
| Note: Full regression models include age, sex, education, BCG vaccination, excess alcohol use, low BMI, COPD, diabetes, TB disease location, positive AFB smear, number of contacts, and DR-TB. All reduced models (shown) include age and sex plus predictor variables with a significance level <0.05. adj. OR=adjusted odds ratio, CI= confidence intervals, DR-TB= resistance to any TB drugs | | | | | | | | | |
