# Supplementary material for: Tuberculosis presentation and outcomes in older Hispanic adults from Tamaulipas, Mexico
Source: Medicine (Baltimore). 2023 Oct 13;102(41):e35458. doi: 10.1097/MD.0000000000035458 (PMC10578661; doi:10.1097/MD.0000000000035458)
Supplement: Supplementary file 3 [file medi-102-e35458-s003.docx]

| **Table S3. Evaluation of age as an effect modifier of the association between the predictor variables listed and adverse TB outcomes** | | | |
| --- | --- | --- | --- |
|  | **Treatment Failure** | **Abandoned Treatment** | **Death** |
| **Predictor Variable** | **p-value** | **p-value** | **p-value** |
| Sex | 0.581 | 0.547 | 0.289 |
| Education | 0.482 | 0.172 | 0.071 |
| BCG Vaccination | 0.420 | 0.727 | 0.800 |
| Excess Alcohol | 0.741 | 0.124 | 0.476 |
| Low BMI | 0.350 | 0.847 | 0.291 |
| Diabetes | 0.406 | 0.298 | 0.518 |
| Extrapulmonary TB^a^ | ꟷꟷ | 0.262 | 0.082 |
| Positive AFB Smear | 0.650 | 0.298 | 0.890 |
| No. of Contacts | 0.535 | 0.641 | 0.060 |
| ^a^ None of the patients with extrapulmonary TB failed treatment | | | |
