# Supplementary material for: Tuberculosis presentation and outcomes in older Hispanic adults from Tamaulipas, Mexico
Source: Medicine (Baltimore). 2023 Oct 13;102(41):e35458. doi: 10.1097/MD.0000000000035458 (PMC10578661; doi:10.1097/MD.0000000000035458)
Supplement: Supplementary file 4 [file medi-102-e35458-s004.docx]

Note: Analysis of each variable includes all 1035 older adults (OA) TB patients except when indicated in parenthesis. Column data is n(%) for categorical variables and mean (standard deviation) for the continuous variable, No. of Contacts. DR-TB= resistant to any of the TB drugs tested (i.e. isoniazid, rifampin, pyrazinamide, streptomycin, ethambutol)

^a^ Score test for trend of odds was calculated for categorical variables and the nonparametric test for trend across ordered groups, an extension of the Wilcoxon rank-sum test, was used for comparing number of contacts across years

^ⴕ^ Trend direction with respect to increasing years is indicated by arrows preceding significant trend p values
